# A new species of *Litopeltis* Hebard, 1920 from Rio de Janeiro, Brazil (Blattodea, Blaberidae, Epilamprinae) with a key to males and geographical distribution of the remaining species of the genus

**DOI:** 10.3897/zookeys.420.7555

**Published:** 2014-06-25

**Authors:** Leonardo de Oliveira Cardoso Silva, Sonia Maria Lopes

**Affiliations:** 1 Departamento de Entomologia, Museu Nacional, Universidade Federal do Rio de Janeiro-UFRJ

**Keywords:** Blaberidae, key, *Litopeltis*, Morphology, new species, taxonomy

## Abstract

This contribution describes a new species of *Litopeltis* from Brazil, *L. teresopolitensis* **sp. n.**, which shows similarities with *L. paineirensis* Lopes & Oliveira, 2010 and *L. ribeiropretano* Lopes & Oliveira, 2010. It differs in characters of morphology genitalia and configuration, with the median sclerite bearing microspines on the sclerotic apex. A map showing the geographic distribution of the Brazilian species and a key to males of the other species of the genus are also presented.

## Introduction

**Figure 1.**
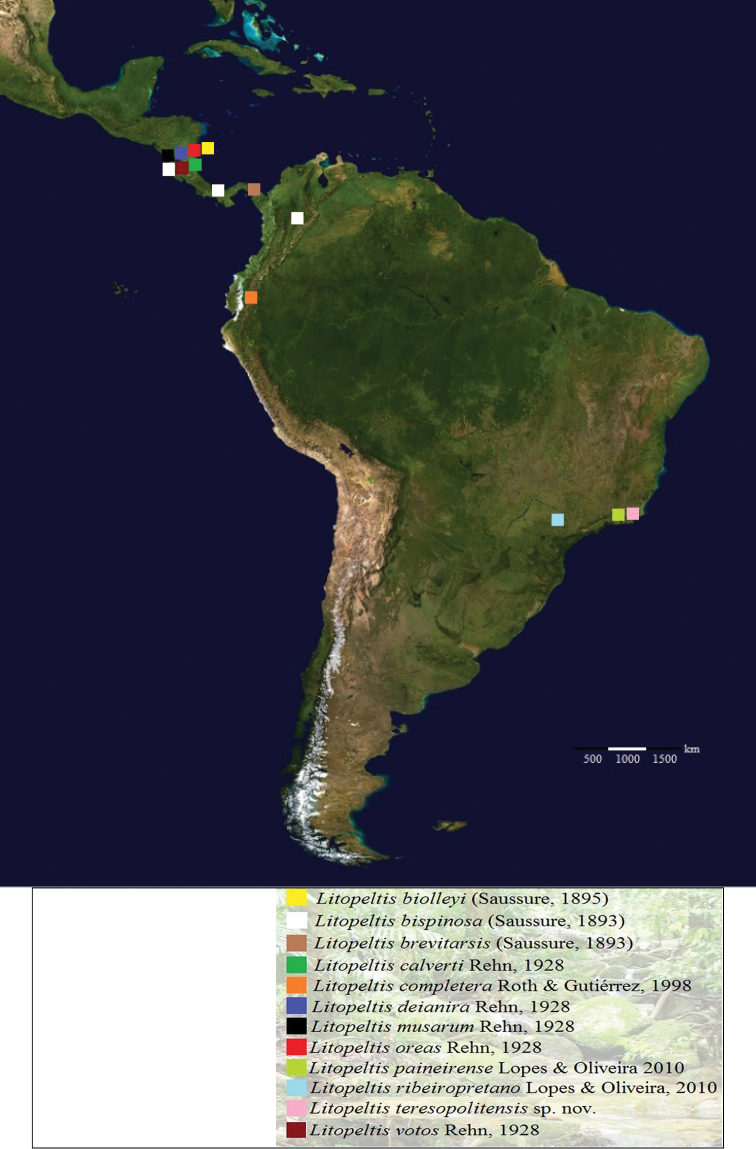
Map of geographical distribution of *Litopeltis* Hebard, 1920.

The genus *Litopeltis* was described by Hebard (1920), based on material from Colombia. Hebard’s decision to include the new genus in the subfamily Perisphaerinae was supported by Rehn (1928), remarking that the male of *Litopeltis* superficially looks much like the Epilamproid genus *Leurolestes*, while the brachypterous females in general resemble species of *Audreia* of the same subfamily. However the position of *Litopeltis* is in the Epilamprinae, differing from *Colapteroblatta* in its size and poorly defined ocelli, as well as from *Mioblatta* in its size, absence of tomentosity, unspotted pronotum and spiked femurs (Roth 1971). The type species is *Litopeltis bispinosa* (Saussure, 1893), previously included in the genus *Calolampra*. Currently *Litopeltis* includes 11 species. Lopes and Oliveira (2010) have determined that in Brazil, members of the genus are present in Rio de Janeiro, São Paulo and Mato Grosso. Beccaloni (2013) recognized 11 species for in the genus, and confirmed its presence in Central America (Costa Rica and Panama) and South America (Ecuador); and Vélez (2008) confirmed the presence of the genus in Colombia (Fig. 1). Thus, the distribution of *Litopeltis* comprises three Neotropical subregions: the Caribbean subregion and the western province of Ecuador (Ecuador) and Chocó (Colombia) and Andean north (Panama), together with the eastern province of Central America and west to the Isthmus of Panama (Panama and Costa Rica); the Amazon subregion, with the province of Pantanal (Mato Grosso); and the subregion of the provinces of Floresta do Paraná (São Paulo) and the Atlantic Forest (Rio de Janeiro) (Morrone 2009). Morrone stated that in the Pre-Quaternary period, the Neotropical biota expanded northward to Central America and southward to the Andean region, which could explain the dispersal of the genus to Central America and to the Amazon and Paraná subregions.

The present contribution provides information about the genus *Litopeltis*, including a key, and a description of a new species. The habitus, head, pronotum and external and internal genitalia with of the holotypes of the Brazilian species and new species were compared, the original descriptions were studied.

## Material and methods

The genital plates were removed after dissection of the posterior part of the abdomen, using traditional dissection techniques, as described by Lopes and Oliveira (2000). After analysis, the genital plates and genital pieces were stored in microtubes containing glycerin and attached to the respective exemplar, a technique developed by Gurney et al. (1964). The terminology for the genitalia and the taxonomic classification follow Roth (2003). The specimens were compared with other specimens of *Litopeltis* deposited in the Blattodea Collection of the Museu Nacional of Rio de Janeiro (MNRJ), and with descriptions in the literature. Digital images of the habitus, pronotum, head and genitalia were taken with a camera mounted on a stereoscope. The holotype is deposited in the collection of the Department of Entomology at the Museu Nacional of Rio de Janeiro (MNRJ). The text was translated by Prof. Solange Garrido and edited by Dr. Janet W. Reid. The map was taken from MAPA DA AMERICA (http://www.mapadaamerica.com/) (Fig. 1).

## Results

### Litopeltis teresopolitensis sp. n.

Taxon classificationAnimaliaBlattodeaBlaberidae

http://zoobank.org/6B0F173A-D1F4-439E-BBF1-90387F3DDBBC

Figs 2–9

#### Typematerial.

Holotype ♂, BRAZIL: Rio de Janeiro, Teresópolis, Parque do Ingá District, III/2013, Schilithz, A. G. col.

#### Etymology.

The name is given for Teresópolis, the collection locality of the holotype.

#### Description.

Dimensions (mm): Male holotype, total length: 20.7; length of pronotum: 4.0; width of pronotum: 4.5; length of tegmen: 17.5; width of tegmen: 4.4.

**Figures 2–9.**
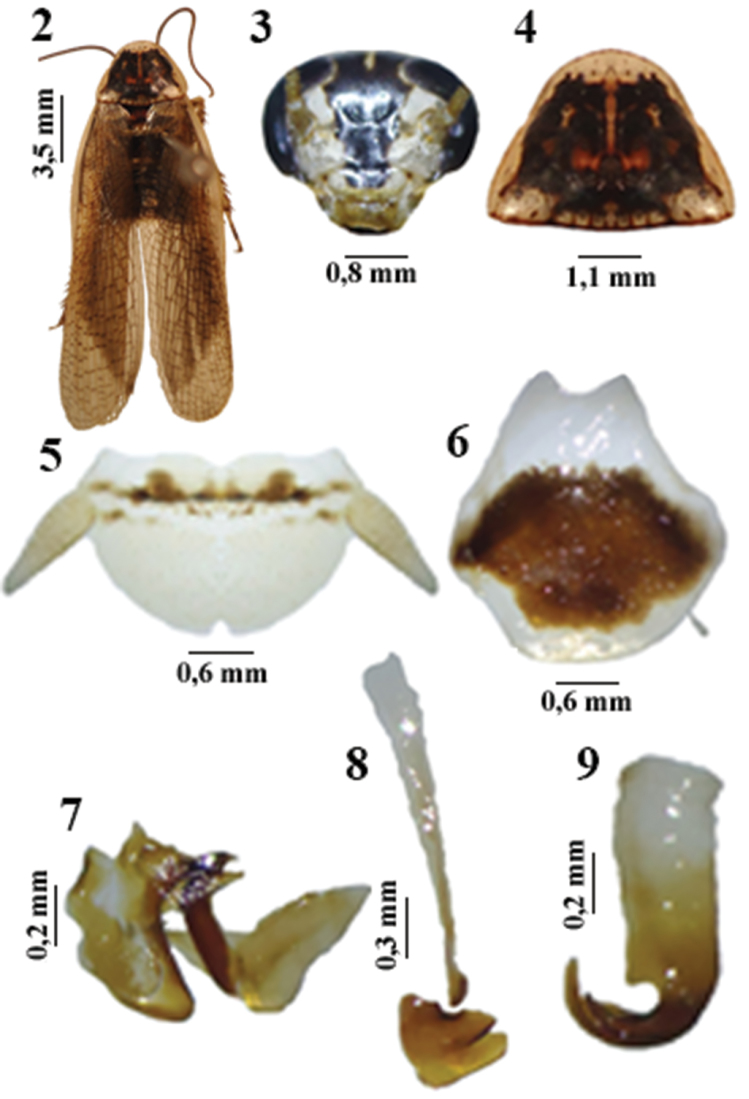
*Litopeltis teresopolitensis* sp. n., male **2** habitus (dorsal view) **3** head (ventral view) **4** pronotum (dorsal view) **5** supra-anal plate (dorsal view) **6** subgenital plate (ventral view) **7** left phallomere (dorsal view) **8** median sclerite (dorsal view) **9** right phallomere (dorsal view).

**Male holotype.** Coloration. General coloring chestnut (Fig. 2). Head with dark eyes, vertex dark with a white vertical line and interocular space in center of the forehead and center of clypeus black (Fig. 3); antennae opaque, first 26 antennomeres glossy, remainder tomentose. Central disk of pronotum with black spots (Fig. 4). Legs with bases of coxae black, spines on tibiaes, dorsal part of tarsus and claws brown, remaining segments of legs, pulvilli and arolium white. Tegmen hyaline, wings with brown veins. Abdomen with dark-brown segments and whitish-yellow lateral margins.

**Head.** Vertex hidden; interocular space ample, subequal to distance between bases of antennal insertions. Antennae tomentose distally, reaching slightly over half of length of abdomen. Ocelli developed. Maxillar palpi tomentose.

**Thorax.** Pronotum transverse, trapezoid, convex with rounded apex and slightly sinuous base. Legs short and robust. Femur I, anteroventral margin bearing three spines in basal half, a row of 21 spines and one longer apical spine in apical half, with a large robust spine on apex; posteroventral margin with row of seven slender spines, ending with two robust spines in apical third. Femur II, anteroventral margin bearing two robust spines, one median, the other apical; posteroventral margin with three to four robust spines. Femur III, antero- and posteroventral margin with two to three robust spines. Large pulvilli present on all tarsomeres; arolia present; claws symmetrical with slight specialization, having eight small rectangular structures.

Wings. Tegmina long, overreaching apex of cerci; marginal field concave and well delineated; scapular field long and narrow with apically oblique arrangement of veins; discoidal field ample and convex, apically widened with a longitudinal arrangement of veins; anal field ample, elongated, with three axillary veins. Hind wings with costal sector having the apices of the veins dilated; apical triangle present; anal field folded fanwise.

**Abdomen.** Absence of tergal modifications. Supra-anal plate short and wide, with smooth median apical indentation; cerci short (Fig. 5). Subgenital plate widened and prominent medially, with acute styli in median apical region of plate (Fig. 6). Left phallomere with median sclerotic structure in shape of an inverted “V” (Fig. 7); median sclerite developed, with microspines on sclerotized apex (Fig. 8); right phallomere hook-shaped (Fig. 9).

#### Diagnosis.

This species appears to be to *Litopeltis paineirensis* Lopes & Oliveira, 2010 (Figs 10–17), which it resembles in the median sclerite (Fig. 16) and the subgenital plate (Fig. 14); and to *Litopeltis ribeiropretano* Lopes & Oliveira, 2010 (Figs 18–25), which has a similar right phallomere (Fig. 25).

**Figures 10–17.**
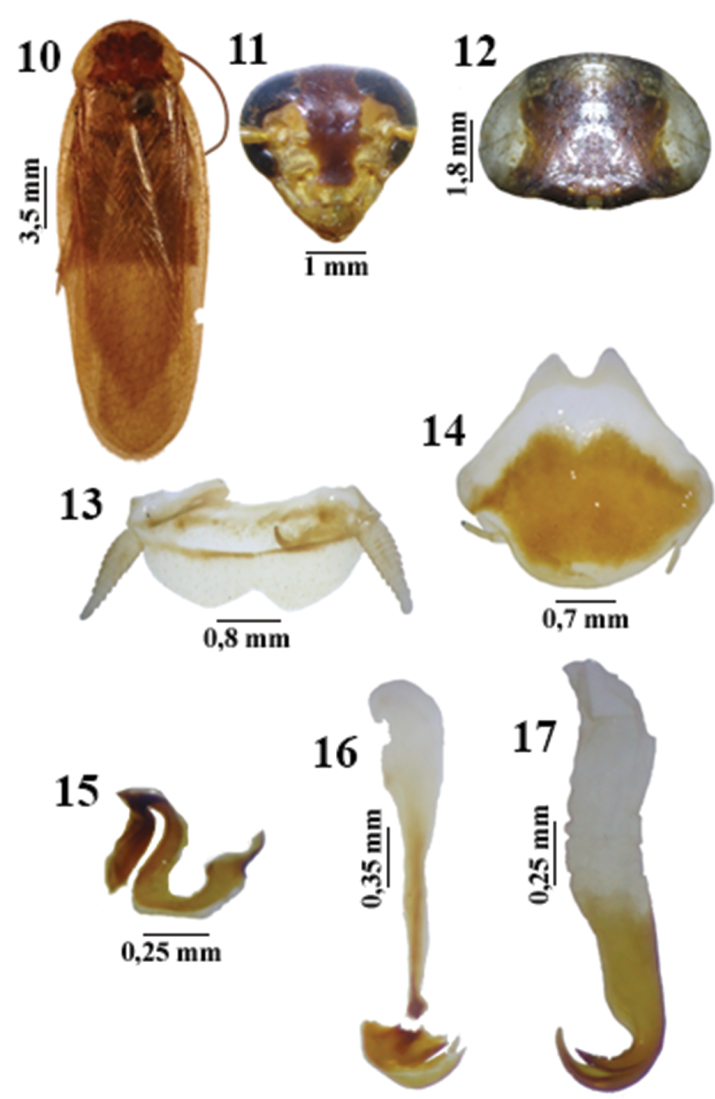
*Litopeltis paineirensis* Lopes & Oliveira, 2010, male. **10** habitus (dorsal view) **11** head (ventral view) **12** Pronotum (dorsal view) **13** supra-anal plate (dorsal view) **14** subgenital plate (ventral view) **15** left phallomere (dorsal view) **16** median sclerite (dorsal view) 17 right phallomere (dorsal view)

**Figures 18–25.**
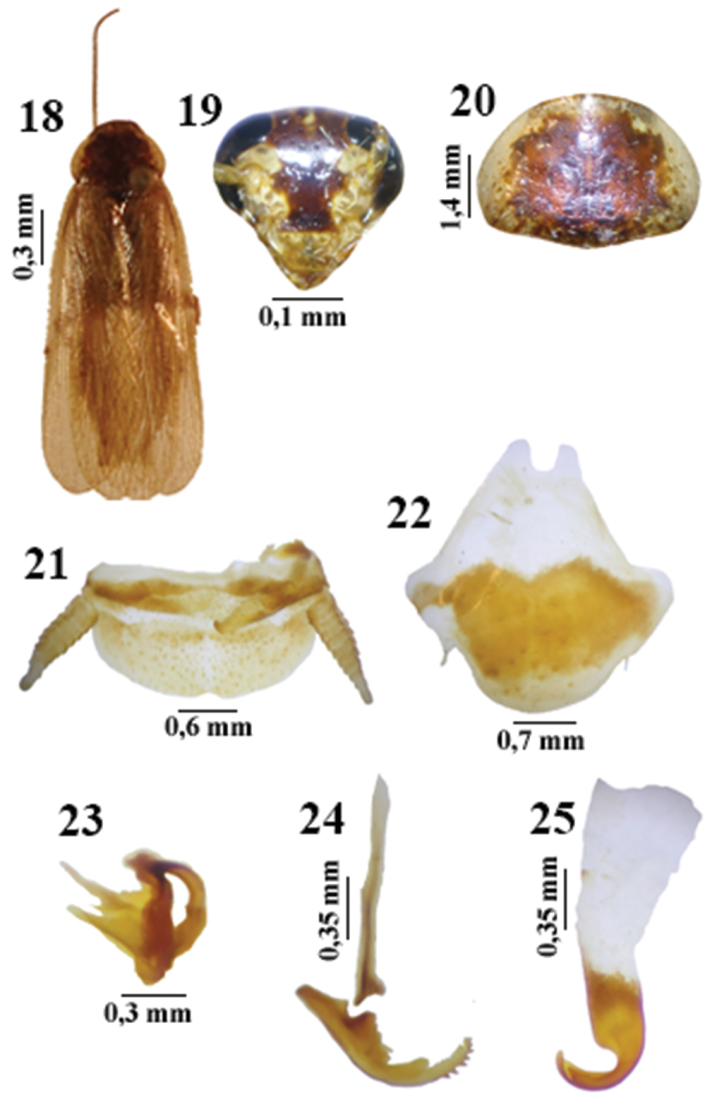
*Litopeltis ribeiropretano* Lopes & Oliveira, 2010, male. **18** habitus (dorsal view) **19** head (ventral view) **20** pronotum (dorsal view) **21** supra-anal plate (dorsal view) **22** subgenital plate (ventral view) **23** left phallomere (dorsal view) **24** median sclerite (dorsal view) **25** right phallomere (dorsal view)

### Key to adult males of the genus *Litopeltis*

The species *Litopeltis brevitarsis* (Saussure, 1893), *Litopeltis compleptera* Roth & Gutierrez, 1998 and *Litopeltis musarum* Rehn, 1928 were not included in the key because they were described from females.

**Table:** 

1	Neotropical species endemic to Brazil	2
–	Neotropical species, however occurring outside of Brazil	4
2	Central disk with nonuniforme spots on pronotum; apex of median sclerite without spike like protuberances (Figs 4, 8, 12 and 16)	3
–	Central disk with uniforme spots on pronotum; apex of median sclerite with spike-like protuberances (Figs 20 and 24)	*Litopeltis ribeiropretano*
3	Right phallomere abruptly tapering pre-apically (Fig. 9)	*Litopeltis teresopolitensis* sp. n.
–	Right phallomere only slightly tapering pre-apically (Fig. 17)	*Litopeltis paineirensis*
4	Total length larger than 15,6 mm	5
–	Total length less than or equal to 15,6 mm	6
5	Dorsal sclerite from median sclerite rounded in the apex and developed, almost reaching the prepuce extension	*Litopeltis bispinosa* (Saussure, 1893) (Figs 39a–41 in Roth 1971)
–	Dorsal sclerite from median sclerite, reduced and thin in the apex, not reaching all prepuce extension	*Litopeltis biolleyi* (Saussure, 1895) (Figs 42–44 in Roth 1971)
6	Dorsal sclerite from median sclerite foliaceous, not reaching the middle of prepuce	*Litopeltis oreas* Rehn, 1928 (Figs 45–47 in Roth 1971)
–	Dorsal sclerite from median sclerite not foliaceous	7
7	Length of pronotum less than or equal to 4,8 mm	*Litopeltis votos* Rehn, 1928
–	Length of pronotum longer than 4,8 mm	8
8	Width of tegmen less than or equal to 6,2 mm	*Litopeltis deianira* Rehn, 1928
–	Width of tegmen longer than 6,2 mm	*Litopeltis calverti* Rehn, 1938

## Supplementary Material

XML Treatment for Litopeltis teresopolitensis
